# In Vitro and In Vivo Models for the Investigation of Potential Drugs Against Schizophrenia

**DOI:** 10.3390/biom10010160

**Published:** 2020-01-19

**Authors:** Oliwia Koszła, Katarzyna M. Targowska-Duda, Ewa Kędzierska, Agnieszka A. Kaczor

**Affiliations:** 1 Department of Synthesis and Chemical Technology of Pharmaceutical Substances, Faculty of Pharmacy, Medical University of Lublin, 4A Chodźki St., PL-20093 Lublin, Poland; koszlaoliwia@gmail.com; 2 Department of Biopharmacy, Faculty of Pharmacy, Medical University of Lublin, 4A Chodźki St., PL-20093 Lublin, Poland; 3 Department of Pharmacology and Pharmacodynamics, Faculty of Pharmacy, Medical University of Lublin, 4A Chodźki St., PL-20093 Lublin, Poland; ewa.kedzierska@umlub.pl; 4 School of Pharmacy, University of Eastern Finland, P.O. Box 1627, FI-70211 Kuopio, Finland

**Keywords:** in vitro models, in vivo models, schizophrenia

## Abstract

Schizophrenia (SZ) is a complex psychiatric disorder characterized by positive, negative, and cognitive symptoms, and is not satisfactorily treated by current antipsychotics. Progress in understanding the basic pathomechanism of the disease has been hampered by the lack of appropriate models. In order to develop modern drugs against SZ, efficient methods to study them in in vitro and in vivo models of this disease are required. In this review a short presentation of current hypotheses and concepts of SZ is followed by a description of current progress in the field of SZ experimental models. A critical discussion of advantages and limitations of in vitro models and pharmacological, genetic, and neurodevelopmental in vivo models for positive, negative, and cognitive symptoms of the disease is provided. In particular, this review concerns the important issue of how cellular and animal systems can help to meet the challenges of modeling the disease, which fully manifests only in humans, as experimental studies of SZ in humans are limited. Next, it is emphasized that novel clinical candidates should be evaluated in animal models for treatment-resistant SZ. In conclusion, the plurality of available in vitro and in vivo models is a consequence of the complex nature of SZ, and there are extensive possibilities for their integration. Future development of more efficient antipsychotics reflecting the pleiotropy of symptoms in SZ requires the incorporation of various models into one uniting model of the multifactorial disorder and use of this model for the evaluation of new drugs.

## 1. Introduction

Schizophrenia (SZ) is a severe mental disorder with a complex pathomechanism. The symptoms of SZ are organized in three classes: positive (e.g. hallucinations, delusions, and thought disorders), negative (e.g. social withdrawal, flattened affect, and anhedonia), and cognitive (e.g. deficits in working memory, poverty of speech, difficulties in attention) [1]. The first concept of SZ was proposed by Emil Kraepelin in 1919 under the name "dementia praecox", who claimed that mental disorders are associated with neuropathology, physiology, and biological chemistry of the brain [2]. According to Kraepelin, different disease syndromes can be associated with different areas of the brain that are more susceptible to pathological stimuli [3]. Eugen Bleuler modified the Kraepelin concept and coined the new name “schizophrenia” for the disease in 1950. He thought that SZ was a heterogenous group of diseases and introduced the concept of “group of schizophrenias” [4,5]. Bleuler elaborated two dichotomous classifications of SZ symptoms: i.e., basic and accessory and primary and secondary symptoms [6]. Basic symptoms are necessarily present in all types of SZ while accessory symptoms may or may not occur. The dichotomy between primary and secondary symptoms is based both on etiology and pathogenesis. Primary symptoms are derived directly from neurobiological abnormalities whereas secondary symptoms are rather psychological reactions that can result from primary symptoms. Moreover, Bleuler considered the alteration of associations as the only symptom which is both basic and primary [6]. Kraepelin and Bleuler speculated that brain dysfunction is responsible for SZ symptoms, in particular that frontal and/or temporal lobe dysfunctions are linked with cognitive abnormalities [7]. However, they were not able to prove this hypothesis with the tools of their era. In 1972, Plum stated that “schizophrenia is a graveyard for neuroanatomists” as no single pathological lesion could be found. On the other hand, Mirsky concluded in 1969 that there was evidence of brain dysfunction in SZ from cognitive, neurological, and electroencephalographic examinations [7]. This was further confirmed by Seidman in 1983 who searched for the neurobiological basis of SZ, in an era when computer tomography (CT) and positron emission tomography (PET) began to be applied to patients with SZ. The SZ concept has changed significantly over the last 30 years, as SZ has been increasingly considered as a neurodevelopmental disease [7].

In spite of numerous attempts to provide reliable diagnostic tools of SZ, it remains defined by the identification of behavioral disorders, in particular psychotic symptoms. Several putative biological markers associated with this disorder have been identified [8], including metabolic biomarkers, neurocognitive dysfunction, brain dysmorphology, and neurochemical abnormalities. However, none of these markers have the appropriate sensitivity and specificity in diagnostic tests. Genetic and association studies targeting multiple loci and genes have also failed to fully demonstrate that any particular gene variant or combination of genes underlies the formation of SZ [4]. Genetic factors are rather thought to predispose to the development of this disease when accompanied by unfavorable environmental factors such as long-lasting stress or multiple stressors simultaneously.

There are significant limitations of current antipsychotics used to treat SZ, including classical first-generation drugs such as chlorpromazine but also second- and third-generation drugs, olanzapine, risperidone, clozapine, aripiprazole, brexpiprazole, cariprazine, and many others. First of all, they are mainly efficient against positive symptoms. Secondly, epidemiologic studies have demonstrated that approximately 50% of the patients with chronic SZ do respond to treatment and have a favorable outcome in terms of functionality measures [9,10]. Finally, current antipsychotics exert severe side effects such as Parkinson-like extrapyramidal symptoms (in particular first-generation antipsychotics), and metabolic side effects including obesity, diabetes etc. (in particular second-generation antipsychotics). The side effects of drugs used to treat SZ reduce patient compliance with the drug regimen, which often leads to recurrence of the disease. There is no doubt that novel antipsychotics are urgently needed and the methods to investigate them in the early, preclinical, and clinical phase of development have to be improved.

In the light of above, the aim of this review is to summarize shortly current concepts and hypotheses of SZ and to discuss current in vitro and in vivo models to study SZ and antipsychotics. Special attention is given to advantages and limitations of the presented approaches as well as their applicability to model the disease which fully manifests only in humans and which is often treatment-resistant with available drugs.

## 2. Hypotheses and Concepts of SZ 

### 2.1. Neuroanatomy of SZ

Over the last 30 years technologic achievements in functional brain imaging have supplied exciting and informative insights into the functional neuroanatomy and neurochemistry of SZ [11]. Modern imaging methods also enable the study of abnormalities on the receptor level. PET and single photon emission computed tomography (SPECT) have been applied to measure dopamine-related parameters using radioligands which bind to receptors, transporters, or other target molecules [12], and to trace a metabolic pathway [13].

In schizophrenic patients, dilation of the brain cavities, volume changes of some nuclei basales, and changes in the hippocampus were observed. SZ patients often have an enlarged ventricular system, third ventricle enlargement, temporal anomalies of the upper comers [14], and irregularities within the frontal and temporal lobe structures [15]. One approach to understanding of the lesions is assessment of changes occurring in healthy siblings, used as so-called endophenotype [14]. Most often the changes in healthy siblings are less intensive than in suffering ones but still more visible than in healthy individuals.

According to many preclinical studies, anatomical and physiological changes are frequently observed in the hippocampus in patients with SZ. A structural decrease in hippocampus volume was observed in post-mortem and imaging studies, in particular in patients with the first episode. In addition, the greater reduction of hippocampal volume was observed in chronic patients vs early illness patients [16]. In addition to anatomic changes, people with SZ also have differences in the activity of the hippocampus. This structure plays a major role in declarative memory. which leads to disability during memory tasks in patients with SZ. Moreover, numerous cellular and molecular changes have been observed in people suffering from this disorder. People with SZ have reduced stem cell proliferation in the dentate gyrus. Next, SZ patients demonstrate a reduced number of synapses between the hippocampal mossy fibers and pyramidal neurons, which is associated with changes in synaptic plasticity markers [17,18]. All above findings confirm the dysfunction of the hippocampus structure in SZ. 

The prefrontal cortex is also involved in the pathology of the disorder. The region called the dorsolateral prefrontal cortex is responsible for the working memory, which is disturbed in the disease. Therefore, it is considered that abnormal prefrontal cortex activity contributes to cognitive dysfunction observed in patients with SZ. Interestingly, the prefrontal cortex and hippocampus are connected neuronal systems and dysregulated prefrontal and hippocampal activity may underlie some of the symptoms of SZ [18].

### 2.2. Disturbances in Neurotransmission in SZ

**Figure 1:**
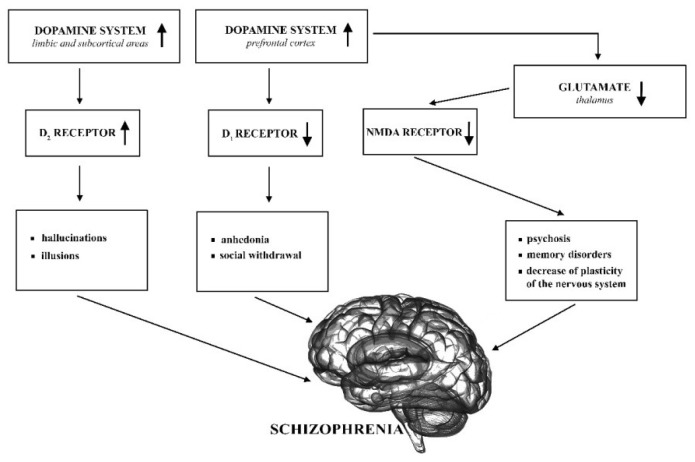
Dopaminergic and glutamatergic system abnormalities in schizophrenia (SZ). NMDA: N-methyl-D-aspartate.

Dopaminergic hypothesis of SZ supplemented by the glutamatergic hypothesis are still the most important regarding the neurobiology of this disease (see Figure 1) [19]. 

The original dopaminergic hypothesis claims that overactivity of the dopamine system causes schizophrenic symptoms, in particular hallucinations and delusions. On the other hand, anhedonia and social withdrawal result from reduced activation of the D_1_ receptor [20]. The most commonly accepted hypothesis today is aberrant silent hypothesis which underlies the symptoms of SZ with abnormalities in the dopaminergic neurotransmission. This hypothesis is on the incentive salience hypothesis [21] which proposes that the mesolimbic dopaminergic neurotransmission is critical in the attribution of salience governing attention and affects decision making and functioning [19,22]. The aberrant salience hypothesis suggests that the attribution of salience is hindered by excessive dopamine firing in psychosis, while in healthy individuals, dopamine is responsible for mediating contextually appropriate saliences [19,23].

Another possible mechanism that can be involved in SZ is dysfunction of the glutamatergic system that affects synaptic plasticity and—among others—N-methyl-D-aspartate (NMDA) receptor signaling [24]. These receptors play an important role in neurotransmission and plasticity, and their underactivity leads to morphological changes that can cause structural changes in the brain and chronic psychosis [25]. A significant importance for glutamatergic system in SZ was first proposed about 30 years ago. It was based on the observation that the psychotomimetics, phencyclidine (PCP), and ketamine induce psychosis and cognitive deterioration similar to SZ by antagonizing neurotransmission at NMDA-type glutamate receptors [26]. The combination of NMDA hypofunction and presynaptic dopamine dysfunction can provide a very good explanation for most clinical aspects of SZ [27]. Further support for the glutamatergic hypothesis of SZ is supplied by the investigated possibility to use ligands of metabotropic glutamate receptors (mGluRs) [28,29], in particular their positive allosteric modulators [30] to alleviate SZ symptoms.

Other neurotransmitter systems are also involved in SZ. In particular, changes in serotoninergic neurotransmission may lead to negative and cognitive symptoms of the disease [19,31]. The serotonin hypothesis of SZ is based on the reports about the hallucinogenic drug lysergic acid diethylamide (LSD) and its mechanism of action linked to serotonin [32]. It is well known that in particular second-generation antipsychotics, such as olanzapine or risperidone, display better affinity to the serotonin 5-HT_2A_ receptors than to the dopamine D_2_ receptors, which is also a determinant of their atypicality. Atypical antipsychotics exert fewer extrapyramidal symptoms and are more efficient in the treatment of negative and cognitive symptoms than the classical first-generation drugs. Antagonism of 5-HT_2A_ receptor and agonism or partial agonism of 5-HT_1A_ receptor are favorable for antipsychotic activity of novel compounds [33].

Other aminergic neurotransmitter systems, such as adrenergic, muscarinic and histaminergic also have some role in SZ. They are particularly useful for the treatment of cognitive disturbances [34,35,36,37].

It has been also reported that GABA-ergic (γ-aminobutyric acid) neurotransmission is altered in SZ [38]. The GABA system is one of the main inhibitory systems in the brain and is capable of diminishing the dopaminergic system neurotransmission. It has been found in clinical studies that GABA receptor agonists alleviate some SZ symptoms [39]. Further studies are needed to determine if ligands acting on the inhibitory GABA system may supply a sensory filter for the brain in flames as it was suggested.

Nicotinic receptors are also important for the pathomechanism of SZ [40] as most of the schizophrenic patients smoke [41]. In particular, positive allosteric modulators of some subtypes (e.g. α7) of the nicotinic receptors may be useful new drugs for SZ [42,43].

Finally, there are also other concepts and hypotheses of SZ, including the cannabinoid hypothesis of SZ [44]. There are also reports about the role of inflammation [45,46] and oxidative stress [47] in this disease.

### 2.3. Genetic Origins of SZ

Despite many years of research, the knowledge of the etiology and pathogenetic development of SZ is still limited. First reports about heritability of SZ are derived from family studies. It is estimated that if both parents of the patient suffer from SZ, the probability that the patient will develop SZ is about 50%. Studies involving identical twins indicate 60%–90% heritability [48,49,50,51]. These studies tried to indirectly assessed the heritability of SZ, however newer investigations enable direct genomic approaches (comparative genomic hybridization; single nucleotide polymorphism (SNP) chips; next-generation sequencing (NGS); genome-wide association studies (GWAS), and the clustered regularly interspaced short palindromic repeats-associated nuclease 9 (CRISPR/Cas9) genomic editing system) [52]. These modern approaches also have their shortcomings, which are mostly consequences of the complexity of the pathomechanism of SZ [52].

The first specific gene which was associated with SZ is disrupted-in-schizophrenia 1 (DISC1) [53]. It was demonstrated that a balanced translocation disrupting this gene co-segregated with SZ; however, the mechanism of it and linkage to SZ symptoms remains unclear. Since 2014 when GWAS produced a number of hits, some genetic changes have been associated with SZ comprising complement component 4 (C4), neurexin 1 (NRXN1), RNA-binding motif 12 (RBM12), and SETD1A [54]. It should be also stressed that copy number variants (CNVs) have been strongly implicated in SZ risk, as previously reviewed [54,55].

It is assumed that genetics is one of the keys factor leading to the emergence of SZ; however, the occurrence of the disease depends also on environmental stress and neurodevelopmental factors. Genetics may predispose a patient to the disease; however, genetic factors solely do not lead to the development of SZ. Whereas proofs from twin studies suggests a strong heritable component, few individual loci have been found in genome-wide screens, which suggests a role for epigenetic effects [56]. It is supposed that large numbers of weakly acting loci may cumulatively increase disease risk, including several mapping to epigenetic pathways and contributing to the complexity of the disease [56].

### 2.4. Neurodevelopmental Hypothesis of SZ

According to the neurodevelopmental hypothesis, the etiology of SZ includes pathological processes of the central nervous system that take place during fetal life and then in the perinatal period and early childhood [57,58]. A very important phenomenon in the neurodevelopmental hypothesis is excessive pruning of synapses and loss of brain plasticity. Much evidence indicating the neurodevelopmental origin of SZ has been found in in vitro, in vivo, and post mortem studies which reveal the quantitative loss of neurons, changes in the size of some brain structures, and cytoarchitectonic changes in cells. The first model which took into account neurodevelopmental abnormalities was termed the “single hit model” which was followed by the “second hit model” and was finally developed into the “multiple hit model” [59]. The multiple hit model best represents complex genetic, social, and environmental interactions that have been explored as contributing to the development of SZ [59].

## 3. In Vitro Models of SZ

Advances in the field of stem cell genetics and biology make in vitro research important for understanding central nervous system diseases. The use of in vitro techniques with a selected cell model makes it possible to carry out molecular, developmental, and pathophysiological studies with high credibility. In this type of research, the selection of an appropriate in vitro model is very important, but because of the large access to many types of cell lines this task is difficult [60].

One of the important models for studying SZ are nerve cell cultures. In 1977, Banker and Cowan obtained for the first time the primary culture of nerve cells from the rat hippocampus. This line turned out to be an ideal model of neural cultures used to date [61]. Some populations of nerve cells can be obtained directly from living people and cultivated as primary cultures. However, ethical views limit the use of primary human brain tissue for research. Due to the fact that the primary nerve cells are also difficult to obtain (especially human neurons), cell lines which can be differentiated into neural cells have been used in in vitro research. What is more, stem cells have also found widespread use in research. The presented cell lines are very good models for the study of intracellular mechanisms as a result of drug administration, for molecular research and genetic susceptibility as well [62,63].

**Figure 2:**
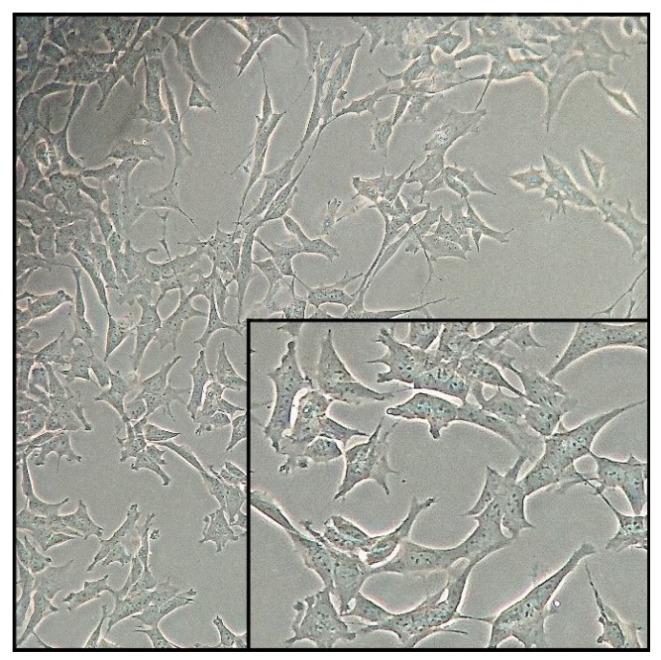
SH-SY5Y cells.

Continuous cell lines established from human tumors are widely used as in vitro models. Some of the tumor cell lines have the characteristics of nerve cells. Currently, the most frequently used cell line with neuronal features is the SH-SY5Y line (Figure 2)—cells of the human neuroblastoma tumors. The SH-SY5Y cell line was first obtained in the seventies of the last century from the bone marrow of a patient with a neuroma. Cells show biochemical and functional characteristics of neurons, which can include: neurite growth, synthesis of neurotransmitters, and activity of neuronal enzyme markers. An additional advantage of this cell line is the expression of specific proteins and isoforms of proteins that are not naturally present in primary rodent cultures [63]. Thanks to these properties, this line is the most frequently used in vitro model for Parkinson’s disease, SZ, Alzheimer’s disease and Huntington’s disease research as well as for the study of the mechanisms of antidepressants and antipsychotics. Furthermore, SH-SY5Y cells can be differentiated using substances such as retinoic acid, resulting in different phenotypes of nerve cells. Non-differentiated cells proliferate continuously and are characterized by the presence of immature neurons. Often undifferentiated SH-SY5Y cells are considered to be immature catecholaminergic neurons which cannot be counted among their positive attributes. After application of differentiation-inducing substances, the cells exhibit characteristics similar to primary neurons. There is a decrease in the rate of proliferation and an increase in c neuron-specific enolase (NSE) activity, which is one of the tumor markers. An important advantage of SH-SY5Y cells can be differentiation into neuronal phenotypes such as dopaminergic, adrenergic and cholinergic phenotypes. One of the most commonly used methods of inducing cell differentiation is the addition of retinoic acid (RA) to the culture medium [64,65].

In addition to the numerous advantages of the SH-SY5Y cell line used in the study of central nervous system diseases, neurotoxicity, and neuroprotection, several disadvantages should be mentioned. The described cell line was derived from tumor cells, which underwent numerous neoplastic transformations as a result of multiple passages. Therefore, this line has a diploid number of chromosomes and is characterized by unlimited growth time. To sum up, due to numerous genetic aberrations, SH-SY5Y cells may change their current function. In addition, cells show little sensitivity to neurotoxins and are not synchronized, therefore not all have markers that are characteristic of mature neurons. 

Another important model to study the mechanisms of central nervous system diseases are stem cells. Stem cells must have two main features. The first feature is the ability to proliferate in the unlimited manner. This characteristic also applies to the cancer cells described above, which are divided in an uncontrolled manner, while stem cell division is strictly regulated. The second very important feature is the ability to share and differentiate into mature cells. There are two main classes of stem cells used as a model for in vitro studies of neurodegenerative diseases: pluripotent, which can become any cell in the body, and multipotent, which can turn into any type of cell but a more limited population. 

Multipotent stem cells are a very good option for clinical use. Thanks to their properties, they can become any cell type found in the human body. Multipotent cells together with the respective compounds differentiate into particular cell lines [66]. Neuronal stem cells (NSCs) are an excellent in vitro model for studying the mechanisms of SZ. Cells occur in the central nervous system in the subgranular zone (SGZ) of the hippocampal dentate gyrus and in the periventricular zone. Neuronal stem cells are characterized by an unlimited ability to self-renew, the ability to differentiate into specialized cells and also have a normal number of chromosomes compared to cancer-derived cells. NSCs provide a very good model for in vitro research, in particular to explore the mechanisms of drugs treatment and to identify susceptibility genes for mental disorders. Additional features of neuronal stem cells are strong plasticity, easy isolation, and in vitro culture, as well as low immunogenicity and high immigration capacity. Neuronal stem cells were used to study pathogenic changes in the expression of the DISC1 gene, which is important in the development of SZ. Application of the NSC model in in vitro studies allows for investigation of the molecular functions of susceptibility genes for SZ [62,66,67].

In addition, induced pluripotent stem cells (iPSCs) are reported as a very useful cell technology that has been applied to cells taken from psychiatric patients. IPSCs can be transformed into all cells in the human body, including central nervous system cells [66]. In particular, skin fibroblasts were taken from SZ patients and healthy controls, “reprogrammed”, and then neurons grew from pluripotent stem cells. Interestingly, neurons from SZ patients presented altered expression of genes involved in signaling of glutamate, cyclic adenosine monophosphate (cAMP) and the WNT protein family, as well as reduced synaptic connectivity and neurite number [68]. IPSCs might be also used to study in vitro neurodevelopmental features of psychiatric disorders. It is also possible to provide a source of models of neurons from individual patients by reprogramming human fibroblasts directly into neuronal cells (i.e., “induced neuronal cells”) [69]. This will help to investigate mechanisms of drug response as well as to study mature cellular pathophysiology of SZ.

Despite the many advantages of application of in vitro models of stem cells, there are several limitations that have to be mentioned. At the moment, current research involving stem cells is based on several samples; it is still necessary to optimize the conditions of isolation and stem cell differentiation. Protocols for induction of stem cells are still in the development phase, so when choosing them as a model for research, we need to take into account not only their advantages but also the disadvantages and the fact that they have not been fully characterized [62].

**Figure 3:**
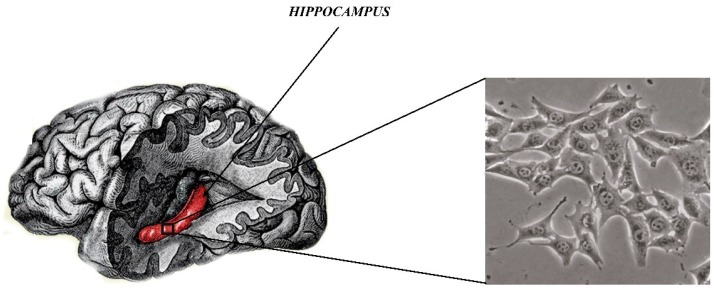
Mouse hippocampal neurons (HT22) cells.

In recent years, a line of mouse hippocampal neurons (HT22, Figure 3) has been used as the main and very good model for in vitro studies of mental diseases such as SZ. More specifically, HT-22 is an immortalized mouse hippocampal cell line subcloned from the HT-4 cell line. The HT-4 cells were originally immortalized from a primary mouse hippocampal neuronal culture [70].

This line is a valuable tool for understanding the molecular and cellular processes important in SZ and other neuropsychological diseases [70]. As it is commonly known, the hippocampus is essential for the proper functioning of the brain mainly to encode and transmit information in the sensory system. Neuropsychiatric disorders are associated with structural and functional abnormalities of hippocampal neurons. HT22 cells can provide a simple way to identify molecules and complex cellular mechanisms in SZ. HT22 cells provide an in vitro model for cytotoxicity studies, mainly cytotoxicity of glutamate, which is an indicator of many neurodegenerative diseases [71]. More specifically, glutamate is a neurotransmitter that is used as a signaling molecule between nerve cells and is mainly responsible for the proper functioning of the brain, including learning and memory. Small levels of glutamate are necessary for the proper functioning of the brain, whereas elevated levels of glutamate can lead to excessive stimulation of nerve cells which will damage cells or activate the intrinsic pathway of apoptosis. Cytotoxicity, which is induced by glutamate, plays a pivotal role in the development of neurodegenerative disorders such as Parkinson’s disease, Alzheimer’s disease, SZ, and depressive disorders. In this regard, inhibition or reduction of glutamate level is known as effective treatment strategy of the above mentioned disorders. However, this is not the case of SZ, which is characterized by the reduced level of glutamate. Moreover, hippocampal neuronal cells are useful for testing drugs for many diseases of the central nervous system [72,73,74]. An additional advantage of culturing HT22 cells is the feasibility of conducting and performing experimental procedures. The hippocampal cells, as a result of numerous passages, retain their cytoarchitectonic properties and are useful to study many aspects of cellular and molecular mechanisms [74]. Available literature data does not show disadvantages of implementation of the mouse hippocampal neurons as an in vitro test model. 

Application of the right in vitro cell model is very important aspect in the drug discovery process. One of the greatest achievements in cell culture techniques was obtaining three-dimensional (3D) culture systems, which are one of the best models for in vitro studies. Until now, in most tests traditional two-dimensional, single-layer cell cultures (2D) on rigid and flat surfaces have been used [63]. Although 2D cultures are routinely used in laboratories around the world, they do not reflect the natural environment of cells. Culture cells on flat media have changed metabolism, gene expression and response to stimulus. Recent studies have shown that 3D cultures more accurately reflect the conditions that prevail in the body, which increases productivity of the cell cultured. Furthermore, these cells have an increased physiological response to bioactive substances compared to two-dimensional cultures. However, the most important difference between 2D and 3D cell culture is the difference in their morphology. In two-dimensional cultures, cells adhere to the substrate only on one side of the cell. However, in the case of three-dimensional cultures, the cells adhere to the substrate with their whole surface. More specifically, the substrate surface mimics the extracellular matrix by formation a tissue scaffold that provides structure for cells to attach and grow [75]. In 3D cultures, a commercial culture medium is used, which includes: collagen, lamins, proteoglycan, entactin, and growth factors. The 3D in vitro model is the best model to study Alzheimer’s and Parkinson’s diseases as well as SZ. Next, three-dimensional cultures are a cell model that provides better interactions between cells and cells and extracellular matrix; 3D systems do not have complex vascular systems that support tissues in vivo (oxidize, provide nutrients, remove waste). Cells in three-dimensional cultures are suitable for this function by diffusion. In the case of larger spheroids, the cells at various depths (from the surface of the spheroid) are differently nourished and thus found in other phases of the cell cycle [75,76]. In contrast to 3D cultures in a two-dimensional monolayer culture, all cells are evenly nourished and oxygenated. In order to get to know all the mechanisms that underlie neurodegenerative diseases one should create and choose a cellular model that best reflects the physiological conditions that prevail in the human brain and three-dimensional cell cultures are an example of it [63,75].

## 4. Animal Models of SZ

### 4.1. Pharmacological Models of SZ

Two main pharmacological models of SZ include the amphetamine model and phencyclidine model. An amphetamine model of SZ, developed in 1950s, is based on the hyperfunction of the dopaminergic neurotransmission in the mesolimbic neural system. However, D_2_ receptor antagonists block amphetamine-induced psychosis, including persecutory delusions and auditory hallucinations that are considered as positive symptoms of SZ. It has been presented that single amphetamine administration enhances spontaneous locomotor activity and stereotyped movements as well. In addition, repeated amphetamine administration induces locomotor activity sensitization and then exaggerating hyperlocomotion (i.e., index that has translational relevance to positive symptoms) related to amphetamine re-challenge after withdrawal [77].

It has also been shown that chronic amphetamine treatment did not induce deficits in social interaction in rats [78]. In addition, amphetamine sensitization may be accompanied by deficits in prefrontal cortex (PFC)-dependent cognitive tasks, such as impairments in reversal learning and the extra-dimensional shift in the attentional set-shifting task [77,79], and reduced accuracy with shorter stimulus duration, as well as an increase in omissions in the five-choice serial reaction time task [80]. However, pretreatment with a low dose of either clozapine or haloperidol prevents the induction of sensitization [81], and both drugs attenuate amphetamine-induced impairment in attention performance [82]. Moreover, repeated amphetamine has no effect on acquisition or retention of spatial visual learning and memory (i.e., hippocampal dependent cognition) determined in the Morris water maze [77]. In this regard, cognitive impairment induced by chronic amphetamine administration is restricted to some PFC-dependent tasks; however, hippocampal function is unaltered.

Increasing evidence indicates that hypofunction of glutamatergic neuronal system has been observed in schizophrenic patients [83]. In particular, administration of an NMDA-type ionotropic glutamate receptor–antagonist, for example ketamine or PCP, induced symptoms (i.e., delusions and hallucinations) observed in patients with SZ [84]. PCP induces positive symptoms of SZ in both acute and stabilized chronic patients. In addition, psychotic symptoms are observed in normal volunteers even after administration of low doses of PCP accompanied by negative symptoms of SZ, such as progressive withdrawal and poverty of speech [85]. Additionally, PCP induces cognitive impairments after acute (low dose) and chronic treatment [85]. In the acute PCP animal model of SZ hyperlocomotion, social withdrawal, and cognition and prepulse inhibition (PPI) impairments are observed [78,86,87,88]. However, chronic PCP treatment is used to more accurately mimic the SZ symptoms in rodents. There are some variations in the subchronic PCP treatment that affect the PCP peak concentration in the brain and may induce some differences in the cognitive paradigm. In particular, differences in subchronic PCP treatment, including PCP dose, gender, and strain as well as period of administration [89].

Typical and atypical antipsychotics (e.g., haloperidol and clozapine) attenuate PCP-induced sensitization that confirms the possible modulation of the positive symptoms of SZ [90]. However, negative symptoms are also observed in rodents, as seen in patients with SZ. For instance, social impairments are induced by chronic PCP treatment in rats [91] and mice [92]. They are reversed by haloperidol and clozapine administration in rats [88], and by clozapine but not haloperidol in mice [92]. It is also known that a subchronic PCP dosage regimen impairs reversal learning in rodents, as observed in SZ patients. A reversal learning task is composed of an initial phase (it requires memory of a previously learned reward), followed by second reversal phase. Animals have to acquire the new strategy when a previously rewarded strategy is blocked. In this regard, animals are required to present flexibility and motivation as well as attention so they can implement a new response and inhibit a response that was previously learned [93]. Novel antipsychotic drugs can attenuate the executive dysfunction that is observed in patients with SZ. For example, acute treatment with clozapine, lamotrigine, and phenytoin [94] but not with haloperidol [95] reversed the acute PCP-induced selective deficit in reversal learning. Treatment with asenapine [96] and sertindole [97] as well as clozapine but not haloperidol and chlorpromazine [98] ameliorated deficits in reversal learning induced by subchronic PCP administration. However, it was also shown that acute treatment with ketamine and dizocilpine (MK-801) i.e., NMDA antagonists, produces a pharmacological deficit in reversal learning [99] as observed in SZ.

It has been shown that cortical function impairment is one of the hallmarks of SZ. Deficits are moderate to severe across several domains, including attention, working memory, verbal learning and memory, and executive functions [100]. Below some tests to study cognitive abilities and impairments are presented.

The prefrontal cortex is responsible for many of our cognitive abilities and executive functions such as the ability to modify behavior in response to the altering relevance of stimuli (i.e., cognitive flexibility). SZ patients poorly performed the intradimensional shift/extradimensional shift (ID/ED) task [101] as well as the Wisconsin Card Sorting Test (WCST) [102]. The rodent version of the ID/ED task and WCST is known as the attentional set-shifting task (ASST) [103]. In the ASST rodents must select a bowl containing a food reward based on the ability to discriminate the media covering the bait or the odor. This test requires animals to initially learn a rule and form an attentional ‘set’ within the same stimulus dimensions. In one ED phase of the ASST test, regarded as the index of cognitive flexibility, the rodent must switch their attention to a new, previously irrelevant stimulus dimension and discriminate between the odors and no longer between the media covering the bait [104]. Rats must perform a series of seven discriminations, including simple discrimination (SD), compound discrimination (CD), reversal 1 (R1), intra-dimensional shift (IDS), reversal 2 (R2), extra-dimensional shift (EDS), and reversal 3 (R3). Administration of NMDA receptors antagonists (e.g., ketamine) induces a broad range of SZ-like impairments, including psychotic-like behaviors as well as negative symptoms and cognitive deficits. More specifically, administration of ketamine for 10 consecutive days produces distinct deficits in the ASST that may be attenuated by antipsychotic drugs [105] and nicotinic receptors ligands as well [104]. It was also demonstrated that selective impairments are found in the EDS phase after subchronic treatment with PCP (for seven days followed by seven days washout period). This deficit was ameliorated by 7-day administration of clozapine and risperidone but not by haloperidol [106].

Novel object recognition (NOR) in rodents is used to study declarative (episodic) memory, one of the several cognitive domains that are abnormal in SZ. NOR evaluates the spontaneous exploratory behavior of rodents. In this test, NMDA receptor antagonists, including PCP, MK-801, or ketamine induce deficits in NOR [107]. In the NOR test two identical objects are exposed to animal for short time (3–10 min) in a restricted chamber without any additional cues that may assist learning and memory. First, the time spent to explore each of two identical objects is recorded (i.e., acquisition). The inter-trial interval should be included, mostly between 3 min and 1–3 h, but not greater than 24 h. During second exposure (i.e., retention trial) the rodent is placed back in the chamber and one of familiar objects is replaced by novel object, in the same place as familiar one. The Discrimination Index (DI), enables discrimination between the new and known objects, i.e., it applies the difference in exploration time for familiar objects, and then divides this value by the total amount of time of exploration of the new and known objects. It is expressed by the formula: (DI) = (Novel Object Exploration Time/Total Exploration Time) – (Familiar Object Exploration Time/Total Exploration Time) × 100. 

It is known that schizophrenic patients show deficits in spatial working memory recognition tasks that are relevant to the NOR paradigm [108]. All atypical antipsychotic drugs (i.e., clozapine, asenapine, olanzapine, quetiapine, risperidone, and sertindole) reverse NOR impairments induced by subchronic NMDA receptor antagonist (e.g., PCP or MK-801) treatment [109].

The Morris water maze (MWM) is known as a visual learning and memory task that depends on coordinated action of neurotransmitter systems and several brain regions [110]. The MWM depends on many cognitive substrates such as learning, working and long-term memory, attention, and retention as well. They all are relevant for the deficits found in schizophrenic patients. In the MWM, a round pool filled with water is defined as testing area. There is also a hidden platform that is submerged below the water surface. The animal learns how to escape by finding the platform, in most cases with the help of visual cues. Alternatively, the platform can be placed in another quadrant, or removed during another phase of the experiment. This way, memory retention and extinction can be investigated. Subchronic treatment with PCP induced a significant learning and memory impairment (i.e., increase in swimming distance to the platform). However, PCP-induced deficits are reversed by second-generation antipsychotics such as sertindole, clozapine, and risperidone [111].

Attention has been also identified as being typically impaired in SZ patients. Attention allows an individual to detect, select, and process relevant stimuli and in the same time is responsible for filtering out unnecessary stimuli from all possible environmental stimuli. The 5-Choice Serial Reaction Time Task (5-CSR) has been developed to evaluate attention (sustained and divided) impairments in rodents [112]. In this test animal has to detect the light stimulus (illumination) presented in one of five apertures of a dark operant chamber. After successful identification (via a nose poke) of the correct illuminated aperture, a reward (food pellet) is delivered and the next trial is initiated. If the animal detects incorrect aperture (without illumination) or does not respond within limited time period, then rodent does not receive food reward and house light is illuminated (during 5 s). If the animal responds during inter-trial interval (i.e., before light stimulus is presented), a time out period is initiated as it is an incorrect response. An acute administration of PCP produces a decrease in percent of correct responding and choice accuracy as well as an increase in the correct latency [113]. However, repeated treatment with PCP induces cognitive impairment that leads to a significant reduction in correct responses and accuracy, and increased premature responding. In addition, it causes a significant increase in correct latency, without any influence on completed number of total trials or latency to collect the food reward. Moreover, chronic administration of clozapine partially ameliorated the performance disruptions that were induced by repeated treatment with PCP, significantly reducing the accuracy impairment as well as the increase in premature responding [113].

It has been reported that negative symptom of asociality (i.e., withdrawal from social contact or lack of desire to have social contact) is a core behavioral feature in SZ [114]. Some literature data reveals that NMDA receptor antagonists, such as PCP, MK-801, and ketamine induce social interaction deficits [112]. In particular, subchronic PCP administration impairs social interaction in adult female rats [115]. Social behavior impairments have been reported in mice during PCP withdrawal for up to 28 days after 2 weeks of treatment [92]. However, there are some discrepancies in social interaction evaluation, for example a significantly increased time in which rats actively engaged in social interaction [116]. This discrepancy seems to be due to the differences in the dose, time between the PCP final injection and the social interaction test that was applied. In particular, increased social activity was observed from 24 h until 6 weeks after the final PCP injection [116].

Social interaction is typically evaluated by placing a pair of unfamiliar rodents into an arena under bright conditions, and the amount of time when animals spend engaged with one another is calculated [117]. To minimize anxiety-related components of social interactions several simple changes were described, such as low lighting conditions, habituation to the arena, close weight-matching of the animals, and ad-libitum food availability [114]. A significant decrease in social interaction by test subjects is mostly interpreted as social withdrawal [78,88]. In a different version of social test an arena is divided into several chambers and the time the rodent spends in the chamber with a caged unfamiliar conspecific and the empty chambers is scored [118]. It reflects social avoidance based on calculated preference for the empty chamber. It has been reported that several brain regions, including the hippocampus, amygdala, prefrontal cortex, and cerebellum have been implicated in both social interaction and negative symptoms of SZ [114].

### 4.2. Genetic Animal Models of SZ

Animal models of disruption can be obtained for almost all human genes [119]. The mouse genome is almost as well characterized as the human and mouse models are cost effective, straightforward to produce, and allow investigation at the molecular, cellular, circuit, and behavioral levels [119]. The disadvantage of rat models is the cost of maintenance and it is not balanced by obvious advantages of rat models. The high-throughput and low-cost models involve zebrafish (*Danio rerio)* and fruit fly (*Drosophila melanogaster*), but obviously these systems are not applicable for reproducing more complex human behaviors [119]. It should be stressed that there are a number of shortcomings of usage of genetic animal models to study neuropsychiatric disorders. Interpreting SZ-like phenotypes in mice, including complex symptoms such as paranoia and delusional beliefs, is challenging: they can only be inferred indirectly from disordered mice behavior, a major limitation of modelling SZ in animals [119].

Two most popular genetic mice models of SZ involve DISC1 mouse models [120,121] and neuroregulin-1 models [121]. 

DISC1 is an intracellular scaffolding protein which possesses a number of interacting proteins, facilitating the formation of protein complexes [121]. The DISC1 gene was found as a risk factor for mental diseases in a Scottish pedigree that displayed a translocation between chromosomes 1 and 11 (q42;q14.3). There are three main types of DISC1 mice models of SZ: haploinsufficiency models, point-mutation models, and transgenic (dominant negative) models.

The chromosomal translocation identified in the Scottish family disrupts the DISC1 gene at intron 8 on one chromosome, whereas this gene on the other chromosome is kept intact [120]. The disruption of the gene can cause loss-of-function, most probably because of nonsense-mediated mRNA decay [120]. Thus, the total result caused by this genetic mutation can be haploinsufficiency [120]. Three variants of this model have been obtained, termed Δ25bp [122], Δex2/3 [123], and Disc1 locus-impairment (Disc1-LI) [124]. In spite of the fact that Δ25bp mice display working memory impairment and Δex2/3 mice are characterized by a higher impulsivity phenotype, these models exhibit no additional behavioral endophenotypes which typically occur in most severe mental illnesses. However, recent detailed studies found abnormalities at the cellular level in these mice which were reviewed elsewhere [120]. It remains an open question as to what extent the haploinsufficiency models can contribute to the understanding of the molecular biology of the disease, in particular in the light of the complex nature of the DISC1 gene.

Regarding point-mutation models based on DISC1 gene, it is worth mentioning that two missense mutation models were obtained, Q31L and L100P, which lead to depressive-like and schizophrenic-like behavioral phenotypes, respectively [125]. Further studies on the L100P model revealed a number of abnormalities, such as increased dopamine function, changed neurexin function, deregulated glycogen synthase kinase-3α (GSK3α) activities in synapses, and abnormalities in interneuron development [120]. It should be stressed that these models are sensitive to the animal housing conditions and subtle factors such as diet might change behavioral phenotypes typical for these models which should be taken into consideration while interpreting data from these models.

An alternative option to point-mutation based models is to express putative dominant-negative (DN) isoforms of DISC1 in mouse brains to obtain transgenic models [120,126,127,128,129]. In such models a number of cellular and anatomical functions is disturbed with consequences for mental disease like animal behavior, including e.g. social withdrawal. There are a few issues which should be considered while using transgenic models to model SZ. Importantly, cell functioning and communication (including astrocytes and oligodendrocytes) are impaired, so these models may be applied to study disease-related impairment at the cellular level. Next, transient disruption of DISC1 functioning during a neonatal period disrupted long-term potentiation/long term depression (LTP/LTD) later in adulthood, which supplies a basis for cognitive disfunction and its delayed onset in mental disorders [120]. This model can also help to link oxidative stress with human mental diseases and can be used to study SZ-related impairments in different neurotransmitter systems.

The second important animal genetic model of SZ is neuregulin-1 based model. Neuregulins are epidermal growth factors which activate ErbB receptor tyrosine kinases, and have significant roles in normal developmental processes, plasticity, and oncogenesis [121]. In the central nervous system the best characterized neuregulin-1 (NRG1) regulates neuronal migration, glial development and differentiation, and neurotransmission and plasticity [121]. It was suggested in 2002 that NRG1 is a candidate gene linked with SZ [130] and down-regulation of neuregulin1/ErbB4 signaling in the hippocampus is critical for learning and memory [131]. As a consequence a number of mice models were constructed to investigate the functional and behavioral consequences of targeting NRG1 and its receptor, ErbB [121]. In a new study Papaleo et al. [132] developed a transgenic mouse model (NRG1-IV/NSE-tTA) in which human NRG1-IV is selectively overexpressed in a neuronal specific manner. They showed that NRG1-IV/NSE-tTA mice display disturbed behaviors relevant to SZ, such as impaired sensorimotor gating, discrimination memory, and social behaviors. Olaya et al. [133] generated transgenic mice with forebrain-driven Nrg1 III overexpression (Nrg1 III tg) and stated that these mice exhibited several SZ-relevant behaviors including social and cognitive deficits as well as impaired sensorimotor gating. Finally, there are also reports about the role of neuregulin-3 in SZ [134] which may also be a base of animal genetic models.

Other mouse genetic models involve modified genes which code the NMDA receptor NR1 subunit [135,136,137], dysbindin [138,139], and reelin [140,141] (see [142] for review).

### 4.3. Neurodevelopmental Models of SZ

According to human epidemiologic studies infection, malnutrition, or hypoxia in the fetus are the factors which can contribute to the development of SZ. In this context, neurodevelopmental animal models of this disease have been generated where the exposure to harmful prenatal factors was highly controlled [142]. Among neurodevelopmental and lesion models, the prenatal polyI:C injection model, the prenatal methylazoxymethanol acetate (MAM) injection model, the prenatal lipopolysaccharide (LPS) model, and the neonatal ventral hippocampal lesion model will be shortly characterized.

PolyI:C is a synthetic analogue of double-stranded RNA. Prenatal injection of PolyI:C to the pregnant rodent dams results in SZ-like behavior of their adolescent offspring. The observed changes include enhanced locomotor activity as a reaction to psychostimulant (but not increased spontaneous locomotor activity), impaired PPI in auditory startle responses, selective impairment in non-spatial memory processing, social withdrawal, and anhedonia [142]. It is worth mentioning that pretreatment with antipsychotic or antidepressive drugs reversed these behavioral changes in this model by decreasing positive and cognitive symptoms. Less evidence is available regarding the drug efficacy for negative symptoms in this model.

MAM is a neurotoxin which administered at a gestational point of time to rat dams interferes with behavioral phenotypes in their offspring. The behavioral changes include increased spontaneous locomotor activity and locomotor sensitivity to the psychostimulant and cognitive and negative symptoms, such as the reduction of social interaction before puberty and deficits in PPI, spatial learning, and cognitive function after puberty [142]. The literature concerning the efficacy of antipsychotics in this model is rather limited. Gill et al. [143] investigated the effect of haloperidol and subsequent response to a novel α5 gamma-aminobutyric acid (GABA(A)) receptor-positive allosteric modulator (α5PAM), on the activity of the dopaminergic system in the MAM neurodevelopmental model of SZ. They found that MAM rats withdrawn from haloperidol exhibited reduced spontaneous dopaminergic system activity and increased locomotor sensitivity to amphetamine compared with control; however they was unresponsive to α5PAM treatment. Neves and Grace [144] reported that modulators of α7 nicotinic receptor reverse the hyperdopaminergic tone in the MAM model of SZ. Thus, MAM model of SZ causes a disturbance in the dopaminergic neurotransmission in accordance with the dopaminergic hypothesis of SZ. It is also important to mention that the MAM model of SZ involves to some extent negative symptoms of the disease and may be used to search for novel drugs efficient against the negative symptoms.

Bacterial infections during pregnancy can contribute to the development of SZ in the offspring. This is the basis of prenatal LPS model of SZ which involves prenatal exposure to a bacterial endotoxin, LPS, in rodents. LPS binds to TLR4 (toll-like receptor 4) in immune cells, and induces a systemic innate immune response similar to PolyI:C [145]. LPS mice exhibit numerous anatomical and cellular abnormalities, in particular in the dopaminergic system, which manifest as behavioral endpoints (positive, negative, and cognitive symptoms). Wischhof et al. [146] reported that some of the prenatal immune activation effects are sex-dependent, which indicates the problem of gender differences in animal model of SZ. Moreover, it has been also postulated that maternal bacterial infections have additive effect with malnutrition on the development of SZ as studied in the LPS model in the conditions of iron deficiency [147].

As has been demonstrated in post-mortem and neuroimaging studies, the hippocampus displays significant structural and functional alterations in patients with SZ [142]. In this context, the ventral hippocampal lesion animal model was proposed. It is based on pathological presence of ventricular enlargement and hippocampal atrophy in individuals with SZ [148,149]. A neonatal lesion of the ventral hippocampus in rodents by microinjection of ibotenic acid (excitatory toxin) induces abnormal behavioral phenotypes after puberty [142], including positive, cognitive, and negative symptoms. Interestingly, the rodents display a higher consumption of sucrose in respective tests, which is a manifestation of changes in the reward system and it is in contrast to the anhedonia in SZ. In addition to the MAM model, the ventral hippocampal lesion animal model can be used to search for drugs against SZ that are efficient to treat negative symptoms.

## 5. Summary of Discussed Models

The summary of the discussed models is presented in Table 1 and Table 2. Table 1 shows examples of application of the discussed in vitro and in vivo models while Table 2 compares advantages and disadvantages of both groups of models.

## 6. Conclusions

In spite of many years of studies, the pathomechanism of SZ is still elusive and the treatment of this disease is far from perfect. Novel antipsychotics, efficient against not only positive but also cognitive and negative symptoms are urgently needed. In order to design and test novel chemical compounds for potential treatment of SZ, in vitro and in vivo models of the disease are required. The difficulty of animal models in case of mental diseases lies in the fact that the disorder manifests fully only in humans and in animals the conclusions are drawn based on their behavior, which can be challenging especially in the case of paranoic thinking or delusion. Nevertheless, the in vitro and in vivo models of SZ are better than currently available in silico approaches and methods of choice before human studies. In order to obtain better effects, the models may be used in combination, which is particularly important to model-treatment-resistant SZ.

## Figures and Tables

**Table 1:** Examples of studies using in vitro and in vivo models of SZ (partially elaborated based on reference [89]).

**In Vitro Model**	**Study**
SH-SY5Y cell line	Study of molecular mechanisms and study of transmission in signaling pathways [150]
Multipotent stem cells	Gene expression studies and pathway dysfunctions associated with mitochondrial metabolism and oxidative stress [151] Analysis of gene and receptor expression, study of neurodevelopmental pathways [152]
Pluripotent stem cells	
HT-22 cell line	Study of biochemical basis of cellular function and disease processes and neurodevelopmental pathways [153]
Three-dimensional culture systems (3D)	Toxicity studies and determination of the biological or biochemical activity of the compounds [154]
**In Vivo Model**	**Study/Effect**
Amphetamine model Phencyclidine model	Locomotor sensitization; increased mesolimbic dopamine response; persistent deficit in prepulse inhibition (PPI); cognitive impairments [77,155] Enhanced mesolimbic dopamine response; no sustained deficit in PPI; reduced social interaction [90,112]
MAM model Neonatal ventral hippocampal lesion model	Spontaneous hyperactivity; amphetamine- and NMDA antagonist-induced hyperactivity; deficits in PPI; cognitive impairment; reduced social interaction [143,156] Amphetamine- and NMDA antagonist-induced locomotor hyperactivity; cognitive impairments; deficits in PPI and social interaction [149,157]
DISC-1 knock-out Neuregulin1 and ErbB4 knock-out	Increased sensitivity to psychostimulants; cognitive deficits; reduced social interaction; depressive-like behavior; deficits in PPI in some mutants [120,125,158] Spontaneous locomotor hyperactivity; social interaction impairment; PPI deficits in Neuregulin1 but not ErbB4 mutants [130,159]

**Table 2:** Advantages and disadvantages of in vitro and in vivo models of SZ (partially elaborated based on reference [89]).

**In Vitro model**	**Disadvantages**	**Advantages**
SH-SY5Y cell line	Genetic aberrations No synchronization	Biochemical and functional characteristics of neurons Expression of specific proteins and isoforms of proteins Ability to differentiate
Multipotent stem cells	Protocols for differentiation and isoallocation conditions are under development	Differentiate into individual cell lines Unlimited self-renewal capacity Correct number of chromosomes Easy isolation and in vitro culture Low immunogenicity High immigration capacity
Pluripotent stem cells		Differentiation into all types of cells
HT-22 cell line	-	Model for glutamate cytotoxicity studies Ease in conducting experimental procedures Preserving cytoarchitectonic properties
Three-dimensional culture systems (3D)	Lack of even nutrition and oxygenation	Increased physiological response to bioactive substances Better interaction between cells and cells and the extracellular matrix
**In Vivo model**	**Disadvantages**	**Advantages**
Pharmacological (phencyclidine and amphetamine models)	There is no current “gold standard” medication to treat all the symptoms that can be used as a positive control (e.g., haloperidol and clozapine should reverse only positive symptoms of this disease) Models should have an appropriate symptoms homology, construct (replicate pathology and theoretical neurobiological response), and predictive validity to the clinical SZ disorder.	Animal models are very valuable preclinical tools used to investigate the neurobiological basis of SZ Resemble “positive-like” symptoms of SZ Resemble negative and cognitive symptoms of SZ by showing altered social deficits and cognitive impairments Resemble altered mesolimbic dopamine function
Neurodevelopmental models		
Genetic models		

## Abbreviations

5-CSR	5-Choice Serial Reaction Time Task
ASST	Attentional set-shifting task
C4	Schizophrenia comprising complement component 4
cAMP	Cyclic adenosine monophosphate
CRISP/Cas9	Clustered regularly interspaced short palindromic repeats-associated nuclease 9
CT	Computer tomography
DI	Discrimination Index
DISC1	Disrupted-in-schizophrenia 1
DN	Dominant negative
GABA	γ-Aminobutyric acid
GSK3α	Glycogen synthase kinase-3α
GWAS	Genome-wide association studies
iPSCs	Induced pluripotent stem cells
ID/ED	Intradimensional shift/extradimensional shift
LPS	Lipopolysaccharide
LSD	Lysergic acid diethylamide
LTD	Long-term depression
LTP	Long-term potentiation
MAM	Methylazoxymethanol acetate
mGluR	Metabotropic glutamate receptor
MK-801	Dizocilpine
MWM	Morris Water Maze
NGS	Next generation sequencing
NMDA	*N*-methyl-D-aspartate
NOR	Novel object recognition
NRG1	Neuregulin-1
NRXN1	Neurexin 1
NSCs	Neuronal stem cells
NSE	Neuron-specific enolase
PAM	Positive allosteric modulator
PCP	Phencyclidine
PET	Positron emission tomography
PFC	Prefrontal cortex
PPI	Prepulse inhibition
R1, R2, and R3	Reversal 1, 2, and 3
RA	Retinoic acid
RBM12	RNA-binding motif 12
SD	Simple discrimination
SGZ	Subgranular zone
SNP	Single nucleotide polymorphism
SPECT	Single photon emission computed tomography
SZ	Schizophrenia
TLR4	Toll-like receptor 4
WCST	Wisconsin Card Sorting Test
